# Effects of 10-Hydroxy-2-decenoic Acid and 10-Hydroxydecanoic Acid in Royal Jelly on Bone Metabolism in Ovariectomized Rats: A Pilot Study

**DOI:** 10.3390/jcm12165309

**Published:** 2023-08-15

**Authors:** Rina Hanai, Hiroshi Matsushita, Akira Minami, Yuki Abe, Rika Tachibana, Kazushi Watanabe, Hideyuki Takeuchi, Akihiko Wakatsuki

**Affiliations:** 1 Department of Obstetrics and Gynecology, School of Medicine, Aichi Medical University, Nagakute 480-1195, Aichi, Japan; 2 Department of Biochemistry, School of Pharmaceutical Sciences, University of Shizuoka, Suruga-ku, Shizuoka 422-8526, Shizuoka, Japan

**Keywords:** bone mineral density, bone strength, fatty acids, 10-hydroxydecanoic acid, 10-hydroxy-2-decenoic acid, ovariectomy, rat, royal jelly

## Abstract

Although previous studies have demonstrated that royal jelly (RJ) may have estrogenic properties and prevent postmenopausal bone loss, the underlying mechanisms are not fully understood. This animal study aimed to investigate the effects of specific fatty acids of RJ, 10-hydroxy-2-decenoic acid (10H2DA) and 10-hydroxydecanoic acid (10HDAA), in ovariectomized rats. Ten-week-old female Wistar rats were divided into the Baseline, Sham, Ovx, Ovx + 10H2DA, and Ovx + 10HDAA groups. Rats in the Baseline group were sacrificed immediately, whereas those in the other groups were subjected to either a sham operation or bilateral ovariectomy. The animals in the Ovx + 10H2DA and Ovx + 10HDAA groups were fed diets containing 10H2DA and 10HDAA, respectively. Twelve weeks after surgery, the rats were sacrificed, and indices of bone mass and bone mechanics were analyzed. Femoral bone mineral density was significantly lower in the Ovx group than in the Sham group (*p* < 0.01). Administration of 10H2DA or 10HDAA did not ameliorate bone loss after ovariectomy. In addition, administration of these fatty acids diminished femur bone stiffness in ovariectomized rats (*p* < 0.01 and *p* < 0.05, respectively). These findings suggest that the favorable effects of RJ may not be exerted solely by 10H2DA or 10HDAA. However, these effects may be exhibited in combination with other RJ constituents.

## 1. Introduction

Honeybee products such as honey, propolis, bee pollen, and royal jelly (RJ) have traditionally been consumed to promote human health worldwide [1,2,3,4]. RJ is a thick milky substance secreted by the hypopharyngeal and mandibular glands of worker honeybees (*Apis mellifera* L.). RJ possesses various physiological and pharmacological activities, including antibacterial, antitumor, anti-inflammatory, antioxidant, anti-hypercholesterolemic, vasodilative, and hypotensive activities [5]. Animal studies have demonstrated that RJ may exert a bone-sparing effect after menopause. Hidaka et al. [6] and Kadafar et al. [7] reported that administering RJ to ovariectomized rats prevented bone loss, albeit not to the levels observed in sham-operated rats. Although Kaku et al. [8] failed to show the positive effects of RJ in preventing bone loss in ovariectomized rats, they demonstrated that RJ mitigated the reduction in collagen crosslinks, a major post-translational modification of collagen that represents an aspect of bone quality and determines the biomechanical properties of bone in the rat tibia. Recently, we demonstrated that RJ did not prevent bone loss but improved bone strength in ovariectomized rats [9]. Additionally, we demonstrated that RJ consumption prevented the decline in femoral bone mass and strength in healthy postmenopausal women in a randomized double-blind, placebo-controlled study [10].

RJ is a mixture of water (50–70%) and various compounds, such as proteins (9–18%), carbohydrates (7–18%), lipids (3–8%), trace minerals (0.8–3%), amino acids, vitamins, and phenols, and its biological activities have been explored in various studies [11]. However, it is not fully understood which of the components of RJ, alone or in combination with others, have beneficial effects on bone. Estrogens play a pivotal role in maintaining balanced bone metabolism in women; thus, estrogen deprivation after menopause leads to net bone loss due to a marked increase in bone resorption by osteoclasts, which is not fully compensated for by the osteoblast-mediated bone formation [12]. Menopausal hormone therapy (MHT) is one of the therapeutic options in the prevention of postmenopausal bone loss and has been shown to significantly lower the risk of hip, vertebral, and other osteoporosis-related fractures in postmenopausal and non-osteoporotic healthy women [13]. We speculated that the bone-sparing effect of RJ may be exerted by its estrogenic activities because previous studies have shown that RJ may have estrogenic properties [14,15]. Although the estrogenic activities of RJ have not been fully elucidated, previous studies have indicated that fatty acids such as 10-hydroxy-2-decenoic acid (10H2DA), 10-hydroxydecanoic acid (10HDAA), and 24-methylenecholesterol may be responsible for its estrogenic activities [11]. Suzuki et al. [16] demonstrated that these compounds activated the estrogen receptor (ER) using a luciferase reporter gene assay. The investigators also demonstrated that these fatty acids stimulated the proliferation of the human breast cancer cell line MCF-7, blocked by concomitant treatment with tamoxifen. In addition, Moutsatsou et al. [17] showed that RJ-derived fatty acids, namely 10H2DA and sebacic acid, stimulate the mineralization of KS483 osteoblastic cells, which is inhibited by ICI182780, an estrogen antagonist.

Based on these findings, we hypothesized that the fatty acids in RJ might have bone-sparing effects after menopause. 10H2DA, also known as the queen bee acid, is the only fatty acid present exclusively in RJ and is regarded as the major indicator of RJ quality [18]. 10HDAA is the saturated counterpart of 10H2DA and is the second most abundant fatty acid in RJ. 10H2DA and 10HDAA constitute 60–80% of the fatty acids in RJ [11]. Previous studies have shown that 10H2DA also exhibits similar physiological and pharmacological properties to RJ, such as antibacterial, antitumor, anti-inflammatory, and anti-hypercholesterolemic activities [18]. Thus, we speculated that the bone-sparing effects of RJ may be attributed to 10H2DA and 10HDAA. Therefore, we conducted this study to investigate if unique and specific fatty acids of RJ, namely 10H2DA and 10HDAA, could recapitulate the results of our previous study, in which RJ did not prevent bone loss but improved bone strength in ovariectomized rats [9].

## 2. Materials and Methods

### 2.1. Diet

In our previous study [9], ovariectomized rats were orally administered with RJ at a dose of 2.5 g/kg body weight per day. Accordingly, we constructed custom pellet diets containing 1.95 g of 10H2DA (Hangzhou Eastbiopharm Co., Ltd., Hangzhou, China; equivalent to 4.0% of RJ) and 0.575 g of 10HDAA (Combi-Blocks, Inc., San Diego, CA, USA; equivalent to 1.2% of RJ) in 1 kg standard rodent chow (MF; Oriental Yeast Co., Ltd., Tokyo, Japan). The rats were estimated to intake 10H2DA and 10HDAA at doses of 100 and 30 mg/kg of body weight per day, respectively.

### 2.2. Animals

Fifty-five female Wistar rats, aged 8 weeks, were purchased from Japan SLC Inc. (Hamamatsu, Japan). Upon arrival, the rats were housed under controlled conditions (room temperature 23 ± 1 °C; humidity 55 ± 5%; 12 h light–dark cycle) with free access to distilled water and MF. The experiments were performed according to a protocol approved by the Animal Ethics Committee of the University of Shizuoka (approval No. 176283).

### 2.3. Protocol

After 2 weeks of acclimation, the animals weighed 158 ± 4 g (mean ± standard deviation (SD)) and were allocated to five groups: Baseline (n = 5), Sham (n = 11), Ovx (n = 15), Ovx + 10H2DA (n = 12), and Ovx + 10HDAA (n = 12). Rats in the Baseline group were anesthetized by intraperitoneal injection of medetomidine hydrochloride (0.375 mg/kg body weight), midazolam (2 mg/kg body weight), and butorphanol tartrate (2.5 mg/kg body weight) and sacrificed immediately. This group was included to provide reference values for skeletal measurements, thereby enabling the determination of changes in skeletal tissue resulting from surgery and aging [19]. The remaining rats were anesthetized and either sham-operated (Sham group) or bilaterally ovariectomized (Ovx, Ovx + 10H2DA, and Ovx + 10HDAA groups) via the dorsal approach. Three days after surgery, rats in the Ovx + 10H2DA and Ovx + 10HDAA groups started on a custom diet containing 10H2DA and 10HDAA, respectively, and the remaining rats in the Sham and Ovx groups were fed a standard diet. After the 12-week treatment period, all rats were anesthetized and sacrificed. Uterine weight was measured to determine whether the bilateral ovariectomy was performed successfully. The right femurs were removed, cleared of soft tissue, wrapped in saline-soaked gauze, and stored at –20 °C for subsequent densitometry and mechanical testing.

### 2.4. Bone Densitometry

The right femur was completely thawed at room temperature and placed in the anteroposterior position on acrylic plates. Ex vivo bone scanning by dual-energy X-ray absorptiometry (DXA) was performed using a Horizon A DXA system (Hologic, Inc., Marlborough, MA, USA) and analyzed using small-animal software (version 13.3; Hologic, Inc.). Bone mineral density (BMD) was calculated as bone mineral content (BMC) divided by bone area (BA). The femurs were divided into three subregions (proximal, mid-diaphyseal, and distal thirds), and the BMC and BMD of each subregion were also evaluated. The coefficients of variation for the scans and standards were <1.0%.

### 2.5. Biomechanical Testing

After bone densitometry of the right femur, a three-point bending test was performed using an MZ-500S bending tester (Maruto Instrument Co., Ltd., Tokyo, Japan) to analyze the biomechanical properties of the rat femurs at Kureha Special Laboratory Co., Ltd. (Iwaki, Japan). Briefly, each femur was placed horizontally on the supports (13 mm span), and the force was loaded to the midshaft at a constant loading rate of 2 mm/min until fracture. The maximum breaking load (N), displacement (mm), stiffness (N/mm), and breaking energy (Nmm) were analyzed from the load–deformation curves.

### 2.6. Statistical Analysis

Data are expressed as means ± SD. All data management and statistical analyses were performed using JMP software for Windows version 12.2.0 (SAS Institute Inc., Cary, NC, USA). The specific effects of Ovx, 10H2DA, and 10HDAA on body and uterus weights were evaluated by comparing values among the Sham, Ovx, Ovx + 10HDA, and Ovx + 10HDAA groups using one-way analysis of variance (ANOVA) followed by Tukey’s honest significant difference (HSD) test. In addition, their specific effects on bone measurements were examined using analysis of covariance (ANCOVA), with body weight as the covariate [9,20], followed by Tukey’s HSD test. Differences of *p* < 0.05 were reported as statistically significant.

## 3. Results

### 3.1. Effects of 10H2DA and 10HDAA on Body and Uterus Weights

The uterus weight and ratio of uterus/body weight of rats in the Ovx group were significantly lower than those in the Sham group, suggesting that the Ovx surgery was performed successfully. However, there were no significant differences in these parameters among the animals in the Ovx, Ovx + 10H2DA, and Ovx + 10HDAA groups.

There were no significant differences in body weights among the four groups at the time of surgery (0 weeks). However, the animals in the Ovx, Ovx + H2DA, and Ovx + 10HDAA groups gained significantly more weight by the end of the experiment, indicating that the body weights of the animals in these three groups were significantly higher than those in the Sham group. The body weight gain and percentage body weight gain of rats in the Ovx + 10H2DA group were significantly higher than those in the Ovx group, although these parameters were not significantly different between animals in the Ovx + 10H2DA and Ovx + 10HDAA groups (Table 1).

### 3.2. Bone Densitometry of the Femur

The right femoral BMC and BMD of the rats are summarized in Table 2. Both the BMC and BMD of the whole femur in the Ovx group were significantly lower than those in the Sham group. When the femur was divided into three segments of equal length, there were significant differences in BMD between the Sham and Ovx groups at the proximal, mid-diaphyseal, and distal thirds of the femur. The BMC values at the proximal and distal ends, sites high in cancellous bone, were significantly lower in the Ovx group than in the Sham group; but this was not the case at the mid-diaphysis, a site high in the cortical bone. The administration of 10H2DA and 10HDAA to Ovx rats had no significant effects on BMC and BMD, regardless of the femur site (i.e., proximal, mid-diaphyseal, and distal thirds).

### 3.3. Biomechanical Testing

The results of the three-point bending tests on the right femur are presented in Table 3. There was a significant difference in the stiffness of the femur among the four groups, and the values in the Ovx + 10H2DA and Ovx + 10HDAA groups were significantly lower than those in the Ovx group. In addition, the value in the Ovx + 10H2DA group was significantly lower than that in the Ovx + 10HDAA group. The groups had no significant differences in the maximum load, displacement, or energy.

## 4. Discussion

Previously, we have demonstrated that RJ administration improves femoral bone strength but does not prevent bone loss in ovariectomized rats [9]. To elucidate which of the components may be responsible for the bone-sparing effect of RJ, the present study aimed to investigate the effects of the unique and specific fatty acids of RJ, namely 10H2DA and 10HDAA, on the indices of bone mass and bone mechanics in ovariectomized rats. As with RJ in our previous study [9], the present study also demonstrated that neither the administration of 10H2DA nor 10HDAA prevented bone loss in the ovariectomized rats. In addition, administration of these fatty acids did not improve but rather diminished femur bone strength in ovariectomized rats.

The effects of specific fatty acids of RJ on bone metabolism have rarely been investigated using a postmenopausal animal model. To the best of our knowledge, only one previous study has examined the effects of 10H2DA on bone loss induced by estrogen deficiency [21]. In a recent investigation, Tsuchiya et al. [21] demonstrated that oral administration of RJ to ovariectomized mice prevented bone loss by inhibiting osteoclastic bone resorption but did not accelerate osteoblastic bone formation. In addition, using in vitro osteoclast differentiation in a monoculture system, the addition of RJ was found to significantly inhibit the osteoclast differentiation induced by stimulation with macrophage-colony stimulating factor (M-CSF)/receptor activator of nuclear factor-κB (NF-κB) ligand (RANKL) and co-culture of bone marrow cells with calvarial osteoblastic cells in a dose-dependent manner. Subsequently, they fractionated RJ and identified 10H2DA as a constituent that suppressed osteoclastogenesis by in vitro osteoclast differentiation system and showed that the oral administration of 10H2DA prevented bone loss in ovariectomized mice in the same manner as RJ. Furthermore, the expression of the genes implicated in osteoclastogenesis and bone resorption was analyzed to investigate the molecular mechanisms of the bone-sparing effect of RJ and 10H2DA, and it was found that RJ and 10H2DA significantly decreased the expression of *Nfatac1*, a key transcription factor of osteoclastogenesis and its downstream elements in osteoclasts.

It is unclear why the findings of this study and our previous study, which exhibited favorable effects of RJ on femoral bone strength [9], failed to corroborate the results presented by Tsuchiya et al. [21]. In the present study, we fed the animals a custom pellet diet prepared before surgery. In contrast, in our previous study and in Tsuchiya’s studies, the diets were administered to animals by gastric gavage soon after preparation [9,20]. Chemical alteration of fatty acids administered to animals is a major concern during research and may affect the research outcome [22]. 10H2DA is an α, β-unsaturated carbonyl compound with low stability and bioavailability, and it has been suggested that processing of RJ, such as lyophilization, tableting, and encapsulation, may possibly attenuate the bioactive effects of 10H2DA. Tsuchiya et al. [21] also demonstrated that the effect of 10H2DA was exerted by its binding to free fatty acid receptor 4 (FFAR4) on osteoclasts, followed by the inhibition of RANKL-induced activation of NF-κB signaling, thereby attenuating the induction of nuclear factor of activated T cells C1, a key transcription factor for osteoclastogenesis. Thus, we speculate that various factors, such as dietary conditions and administration routes, may have diminished the bioavailability of 10H2DA. Antinelli et al. [23] reported that the 10H2DA loss rates in RJ for the 12-month period were 0.1 and 0.2% at −18 and 4 °C, respectively, and 0.4 to 0.5% at room temperature, regardless of its origin, suggesting that the stability and bioavailability of 10H2DA may be preserved when it is administered in the form of RJ.

Tsuchiya et al. [21] also reported that the effect on osteoclastogenesis was greater in the form of RJ than in the form of 10H2DA, which was equivalent to the dose in RJ, suggesting that RJ may contain other anti-osteoclastogenic molecules and exert its function through mechanisms other than FFAR4. We conducted a series of studies investigating the effects of RJ on postmenopausal bone metabolism [9,10] because previous studies have shown that RJ may have estrogenic properties [14,15]. Estrogen exerts many functions via the two nuclear ERs, ERα and ERβ. Both ERs have been detected in all skeletal cell types, including osteoblasts, osteocytes, osteoclasts, and progenitor cells [24]. However, most of the bone-sparing effects of estrogen are mediated via activation of ERα, and it is considered that ERβ activation may not play a major role in bones [24,25,26]. Similar to RJ [9], administration of 10H2DA or 10HDAA did not prevent ovariectomy-induced changes in body and uterine weights, which are attenuated by estradiol and mediated in large part via ERα [27,28]. Suzuki et al. [16] also reported that RJ-derived 10H2DA and 10HDA activated ERβ but did not change body or uterine weights. Based on these findings, we speculated that the bone-sparing effects of RJ may not be exerted, in large part, by the estrogenic properties of 10H2DA and 10HDA. Recently, Ishida et al. [29] investigated the estrogenic activity of RJ using an in vitro reporter assay and an estrogen-responsive reporter mouse model and found that RJ exhibited little or no estrogenic activity. The investigators concluded that the beneficial effects of RJ on human health may not be exerted through ER-mediated genomic actions. They also speculated that the effects of RJ may be mediated by the non-genomic actions of GPR30 (plasma membrane ER) and the subsequent activation of phosphatidylinositol-3 kinase/Akt signaling.

To the best of our knowledge, this is the first study that examined the effects of 10H2DA and 10HDAA on bone strength in ovariectomized rats to date. It is of note that the present study demonstrated that the administration of 10H2DA and 10HDAA diminished femur bone strength, even though it failed to prevent bone loss in the ovariectomized rats. In our previous study that investigated the effects of RJ, RJ also failed to prevent the loss of BMD, although it improved bone strength [9]. Because bone strength is determined by both bone density and bone quality, treatment with RJ, 10H2DA, and 10HDAA may have affected the bone quality of the ovariectomized rats. Although bone quality is not a clearly understood concept, it designates the property of bone that contribute to strength. However, it is not assessed by bone densitometry, encompassing various factors such as bone mineralization degree, hydroxyapatite crystal size and heterogeneity, collagen properties, osteocyte density, and trabecular and cortical microarchitecture [30,31]. Collagen crosslinking is a major post-translational modification of collagen that represents an aspect of bone quality and determines the biomechanical properties of bone [8,32]. Bone stiffness is defined as the reaction force divided by the applied displacement and represents resistance against deformation, and some studies have demonstrated that decreased collagen crosslinking is associated with decreased bone stiffness [33]. Previous studies reported that RJ increased the expression of genes that encode key enzymes for collagen crosslinking in vitro. In addition, Kaku et al. [8] demonstrated that the administration of RJ significantly mitigated the ovariectomy-induced reduction in collagen crosslinks (pyridinoline and deoxypyridinoline) in tibial bone extracts of mice. In contrast, Claassen et al. [34] reported that manipulation of fatty acids in diets influenced free urinary collagen crosslinks, total urinary hydroxyproline, and bone calcium content in young male rats. Based on these findings, we speculated that RJ [9] and RJ-derived fatty acids affected collagen crosslinking differently, resulting in conflicting results in bone strength. Further studies are needed to investigate how RJ and RJ-derived fatty acids may affect collagen crosslinking of bone.

The present study had some limitations. First, as stated above, 10H2DA and 10HDAA were administered to the animals via custom pellets. In our previous study [9], however, RJ was administered using gastric gavage; therefore, we could not fully reproduce the experimental conditions, resulting in low stability and bioavailability of 10H2DA and 10HDAA. Second, we did not investigate the effects of 10H2DA and 10HDAA using different dose settings, which may have resulted in the deleterious effects of 10H2DA on bone strength. Phytoestrogens may exert biphasic dose-dependent effects, stimulate osteogenesis at low concentrations and inhibit osteogenesis at high concentrations in vitro [35]. Low-dose genistein, a phytoestrogen, has been reported to be more effective than in higher doses at preventing bone loss in lactating ovariectomized rats [36]. Third, the group analysis involved a relatively small number of animals, which may hinder the study results. In addition, although we did not detect significant changes in femur BMD, other methods that can evaluate bone microstructures (e.g., bone histomorphometry and microcomputed tomography) may have detected changes that had not been captured by DXA measurements. For these reasons, current results should be interpreted with caution.

## 5. Conclusions

Administration of 10H2DA or 10HDAA did not prevent bone loss but reduced bone strength in ovariectomized rats. These findings suggest that the favorable effects of RJ may not be exerted solely by 10H2DA or 10HDAA. However, these effects may be exhibited in combination with other RJ constituents. Further studies are required to investigate the mechanisms through which RJ and its components exhibit bone-sparing effects after menopause.

## Figures and Tables

**Table 1:** Mean body weights at the time of surgery (0 weeks) and necropsy (12 weeks), body weight gain, and uterus weights in the sham-operated (Sham group), ovariectomized (Ovx group), and ovariectomized rats administered 10H2DA (Ovx + 10H2DA group) or 10HDAA (Ovx + 10HDAA group) for 12 weeks.

	Sham	Ovx	Ovx + 10H2DA	Ovx + 10HDAA
	(n = 11)	(n = 15)	(n = 12)	(n = 12)
Body weight (0 weeks) (g)	162 ± 7	158 ± 7	154 ± 10	160 ± 12
Body weight (12 weeks) (g)	208 ± 9	233 ± 14 ^a^	241 ± 10 ^a^	244 ± 15 ^a^
Body weight gain (g)	46 ± 8	75 ± 10 ^a^	87 ± 12 ^a,b^	84 ± 14 ^a^
Body weight gain (%)	28 ± 5	47 ± 6 ^a^	57 ± 13 ^a,b^	53 ± 12 ^a^
Uterus weight (g)	0.653 ± 0.205	0.104 ± 0.027 ^a^	0.120 ± 0.029 ^a^	0.123 ± 0.029 ^a^
Uterus weight/body weight (%)	0.31 ± 0.10	0.04 ± 0.01 ^a^	0.05 ± 0.02 ^a^	0.05 ± 0.01 ^a^

Data are expressed as means ± standard deviation of the mean. ^a^ *p* < 0.01 compared with Sham, and ^b^ *p* < 0.05 compared with Ovx (analysis of variance with Tukey’s honest significant difference test).

**Table 2:** Bone mineral content (BMC) and bone mineral density (BMD) of the right femur measured by dual-X-ray absorptiometry in the sham-operated (Sham group), ovariectomized (Ovx group), and ovariectomized rats administered 10H2DA (Ovx + 10H2DA group) or 10HDAA (Ovx + 10HDAA group) for 12 weeks.

	0-Week	12-Week			
	Baseline	Sham	Ovx	Ovx + 10H2DA	Ovx + 10HDAA
	(n = 5)	(n = 11)	(n = 15)	(n = 12)	(n = 12)
Femur BMC (g)					
Whole	0.230 ± 0.012	0.329 ± 0.011	0.297 ± 0.017 ^a^	0.296 ± 0.016 ^a^	0.300 ± 0.014 ^a^
Proximal	0.082 ± 0.004	0.125 ± 0.007	0.109 ± 0.007 ^a^	0.108 ± 0.006 ^a^	0.111 ± 0.007 ^a^
Mid	0.052 ± 0.004	0.075 ± 0.005	0.076 ± 0.007	0.073 ± 0.005	0.073 ± 0.005
Distal	0.098 ± 0.008	0.134 ± 0.007	0.116 ± 0.007 ^a^	0.118 ± 0.009 ^a^	0.120 ± 0.007 ^a^
Femur BMD (g/cm^2^)					
Whole	0.192 ± 0.004	0.231 ± 0.005	0.207 ± 0.006 ^a^	0.209 ± 0.005 ^a^	0.207 ± 0.004 ^a^
Proximal	0.193 ± 0.002	0.233 ± 0.006	0.208 ± 0.008 ^a^	0.209 ± 0.007 ^a^	0.208 ± 0.005 ^a^
Mid	0.164 ± 0.006	0.197 ± 0.005	0.186 ± 0.007 ^a^	0.189 ± 0.005 ^a^	0.187 ± 0.005 ^a^
Distal	0.209 ± 0.006	0.252 ± 0.006	0.222 ± 0.005 ^a^	0.224 ± 0.006 ^a^	0.221 ± 0.005 ^a^

Data are expressed as means ± standard deviation of the mean. ^a^ *p* < 0.01 compared with Sham (Sham vs. Ovx vs. Ovx + 10H2DA vs. Ovx + 10HDAA, analysis of covariance with Tukey’s honest significant difference test).

**Table 3:** Mid-diaphyseal maximum load, displacement, stiffness, and energy of the right femurs from the 3-point bending test in the sham-operated (Sham group), ovariectomized (Ovx group), and ovariectomized rats administered 10H2DA (Ovx + 10H2DA group) or 10HDAA (Ovx + 10HDAA group) for 12 weeks.

	Sham	Ovx	Ovx + 10H2DA	Ovx + 10HDAA
	(n = 11)	(n = 15)	(n = 12)	(n = 12)
Maximum load (N)	104 ± 7	106 ± 4	104 ± 6	105 ± 7
Displacement (mm)	0.589 ± 0.054	0.672 ± 0.090	0.647 ± 0.061	0.712 ± 0.118
Stiffness (N/mm)	344 ± 23	344 ± 15	329 ± 20 ^a,c^	339 ± 19 ^b^
Energy (Nmm)	34.4 ± 6.3	42.1 ± 8.9	38.6 ± 7.0	46.0 ± 12.8

Data are expressed as means ± standard deviation of the mean. ^a^ *p* < 0.01 compared with Sham (Sham vs. Ovx vs. Ovx + 10H2DA vs. Ovx + 10HDAA, analysis of covariance with Tukey’s honest significant difference test). ^b^ *p* < 0.05, ^c^ *p* < 0.01 compared with Ovx (Sham vs. Ovx vs. Ovx + 10H2DA vs. Ovx + 10HDAA, analysis of covariance with Tukey’s honest significant difference test).

## Data Availability

The datasets generated and/or analyzed in the current study are available from the corresponding author upon reasonable request.
